# Processing technology as aroma architect: OAV fingerprints decode differentiation and compatibility of key odorants in Fuding Dabai tea via GC×GC-TOF-MS and sensomics

**DOI:** 10.1016/j.fochx.2026.103530

**Published:** 2026-01-13

**Authors:** Panpan Liu, Jia Chen, Lin Feng, Shiwei Gao, Shengpeng Wang, Jinjin Xue, Xueping Wang, Fei Ye, Anhui Gui, Zhi Yu, Pengcheng Zheng

**Affiliations:** a Key Laboratory of Tea Resources Comprehensive Utilization, Ministry of Agriculture and Rural Affairs, Hubei Qingzhuan Tea Engineering Research Centre, Fruit and Tea Research Institute, Hubei Academy of Agricultural Sciences, Wuhan, Hubei 430064, China; b College of Horticulture & Forestry Sciences, Huazhong Agricultural University, Wuhan, Hubei 430070, China

**Keywords:** Tea aroma formation, technology, Odor activity value (OAV), Volatile organic compounds (VOCs), Multivariate analysis, Sensomics

## Abstract

This study systematically investigates the regulatory role of processing technology in the aroma differentiation of Fuding Dabai tea (*Camellia sinensis*). Using an integrated sensomics approach combining quantitative descriptive analysis and GC × GC–TOF–MS, we deciphered aroma formation in green (GT), white (WT), black (BT), and dark (DT) teas. Among 187 volatiles detected, WT exhibited the highest VOC content (2349.42 μg/L) and the most key odorants (30 of 36). Multivariate statistical modeling identified fermentation degree as the primary factor driving aroma divergence, clearly discriminating fermented (BT/DT) from non/light-fermented (GT/WT) teas. OPLS-DA selected 12 marker compounds (VIP > 1), predominantly alcohols and aldehydes. Sweet aroma exhibited strong correlations with benzeneacetaldehyde (*r* = 0.91) and (*E*)-2-hexenal (*r* = 0.92), while aged aroma correlated strongly with (*E,E*)-2,4-heptadienal (*r* = 0.92) (all *p* < 0.001). We demonstrate that processing reconfigures aroma profiles through enzymatic inhibition, oxidative conversion, and microbial fermentation pathways. These results provide a biochemical basis for aroma-oriented optimization in tea processing and establish the superior suitability of Fuding Dabai for white tea production.

## Highlights

• Processing techniques reshape tea aroma through enzyme inhibition-oxidation-fermentation biochemical axis.
• Comprehensive chromatography identified 187 volatile compounds dominated by alcohols and ketones.
• Thirty-five key odorants with odor activity values ≥1 show distinct process-specific distributions.
• Fermentation extent explained 53.3% variance as dominant factor in aroma differentiation.

## 1 Introduction

Tea (*Camellia sinensis*) is a globally celebrated beverage, renowned for its complex flavor profiles and bioactive properties (Sanlier, Gokcen, & Altuğ, 2018; Zhang et al., 2019). Among quality determinants, aroma constitutes a critical sensory attribute governing consumer preference and product differentiation (Zhou, He, & Zhu, 2023). Tea aroma complexity arises from more than 700 volatile organic compounds (VOCs)—including alcohols, aldehydes, ketones, and esters—whose combinatorial ratios define characteristic fragrance types (Zeng, Watanabe, & Yang, 2019; Zhai, Zhang, Granvogl, Ho, & Wan, 2022).

Distinct tea categories exhibit signature VOC signatures. Green tea's “chestnut-like” aroma is attributed to compounds including (*E*)-*β*-ionone, 3-methylbutanal, and linalool (Zhu et al., 2018; Wang, Hua, et al., 2020). Oxidized black tea features floral-sweet notes dominated by linalool, (*E*)-*β*-ionone, and geraniol (Wang, Huang, Deng, & Ning, 2024), while white tea shares key odorants such as (*E*)-linalool oxide (furanoid), geraniol and *β*-ionone (Bao et al., 2025). Post-fermented dark tea displays dynamic profiles: geraniol, benzyl alcohol, and (*Z*)-nerolidol impart early fermentation fruity-sweet notes, whereas linalool, methyl salicylate, and (+)-*α*-terpineol contribute late-phase minty-woody nuances (An et al., 2023). These category-specific aromas are primarily determined by divergent VOC compositions modulated by processing biochemistry.

Aroma development is co-determined by genetic potential (cultivar) and process-induced biochemical transformations. Cultivars establish VOC precursor baselines, while processing parameters (e.g., withering, oxidation, fermentation) enzymatically and thermally modulate volatile formation pathways (Li et al., 2025; Mozumder, Lee, & Hong, 2025). This cultivar-process synergy defines suitability: identical processing of different cultivars—or varied processing of a single cultivar—yields distinct aroma signatures (Qiu et al., 2023). Evidence includes cultivar-discriminatory VOCs (e.g., (*E*)-linalool oxide, geraniol) in Zhenghe white teas (Bao et al., 2025), and varietal influences on “fruity” notes via jasmine lactone in black tea (Li et al., 2022). The Fuding Dabai, a primary raw material for white tea in China, contains high levels of gallic acid, soluble sugars, and amino acids. These serve as aroma precursors, where phenylalanine, accumulated during withering, is notably converted into the floral aldehyde benzeneacetaldehyde via enzymatic pathways such as those involving phenylalanine ammonia-lyase (Chen et al., 2019; Ma et al., 2022). Critically, processing drives biochemical reactions that define final aroma quality (Huang et al., 2022), with identical fresh leaves yielding radically divergent VOC profiles in Pu-erh, white, and black teas due to process-specific reactions (Wang, He, et al., 2025). These transformations alter VOC distributions across chemical classes, with sensory outcomes further modulated by odor thresholds and synergistic/masking effects (Deng et al., 2024).

However, current research often focuses on comparing multiple cultivars under a single processing type, making it difficult to isolate and definitively attribute VOC profile variations to specific process-induced biochemical pathways. While comprehensive VOC analysis is achievable via GC × GC-TOF-MS (Feng, Li, Liu, & Tan, 2025; Yao et al., 2023), the interactive effects of cultivar and processing on category-specific aroma formation remain systematically underexplored. Therefore, a systematic investigation using a single cultivar processed into multiple tea types is crucial to decouple genetic from processing effects, precisely elucidate the reprogramming of aroma biosynthesis pathways by processing, and uncover the full aroma potential of a specific cultivar. This study bridges this gap by characterizing key odorants in Fuding Dabai processed into four tea categories (unfermented green tea, lightly fermented white tea, fully fermented black tea, and post-fermented dark tea). By integrating GC × GC-TOF-MS, sensory evaluation, and odor activity values (OAVs), we elucidate how processing redirects aroma chemistry in a single cultivar. The findings establish a foundation for cultivar suitability assessment and aroma-directed processing optimization.

## 2 Materials and methods

### 2.1 Instruments and reagents

Ethyl decanoate (Sigma-Aldrich, USA); n-alkanes (C7-C28, 99%, Supelco, USA); electronic balance (Beijing Lanjieke Technology Co., Ltd., China); PDMS/CAR/DVB extraction fiber (Supelco, USA); 7890B GC system (Agilent, USA); SSM 1820 modulator (Shanghai Snow View Electronics Technology Co., Ltd., China); time-of-flight mass spectrometer (EI-0620 TOF, Guangzhou Hexin Instrument Co., Ltd., China); CTC autosampler (PAL RSI, Guangzhou Zhida Laboratory Technology Co., Ltd., China).

### 2.2 Sample preparation

Fresh leaves (*Camellia sinensis* cv. *Fuding Daba*; two leaves and a bud) were hand-harvested in June 2024 from a high-altitude plantation (at an altitude of 1100 m; Shennong Qifeng Tea Co., Ltd., Shennongjia Forestry District, Hubei, China). Leaves were processed into green tea (GT), white tea (WT), black tea (BT), and dark tea (DT) using standardized protocols, with three biological replicates per tea type. Processed samples were stored at −80 °C until analysis.

**Fig. 1:**
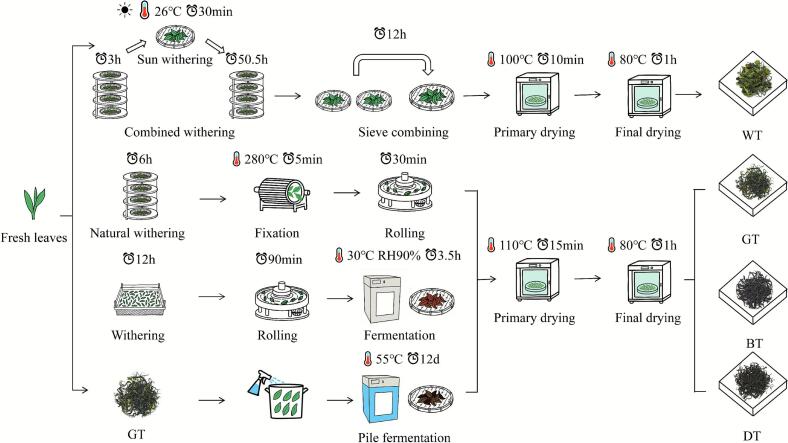
Key processing steps for different tea types made from Fuding Dabai. WT: white tea, GT: green tea, BT: black tea, DT: dark tea, RH: relative humidity. . (For interpretation of the references to colour in this figure legend, the reader is referred to the web version of this article.)

Processing protocols (Fig. 1):

**GT**: Withering (ambient, 6 h) → Fixation (280 °C, 5 min) → Rolling (30 min) → Primary drying (110 °C, 15 min) → Final drying (80 °C, 1 h).

**WT**: Withering (indoor withering, ambient, 2–3 cm thickness, 3 h → sun withering, 26 °C, 30 min → indoor withering, ambient, 2–3 cm thickness, 50.5 h) → Sieve combining (at ∼30% moisture, 12 h) → Primary drying (100 °C, 10 min) → Final drying (80 °C, 1 h).

**BT**: Withering (trough; 12 h to ∼60% moisture) → Rolling (90 min) → Enzymatic oxidative fermentation (30 °C, 90% RH, 3.5 h) → Primary drying (110 °C, 15 min) → Final drying (80 °C, 1 h).

**DT**: GT (from above) → Pile fermentation (28% moisture, 55 °C, 12 d; turned at day 6) → Primary drying (110 °C, 15 min) → Final drying (80 °C, 1 h).

### 2.3 Sensory evaluation

Sensory analysis was performed by a panel of five trained evaluators in strict accordance with the national standard GB/T 23776–2018 (Sensory Evaluation Methods for Tea). Quantitative Descriptive Analysis (QDA) assessed six aroma attributes: fresh, fruity, sweet, floral, chestnut-like, aged. Intensity was scored (0–8 scale: 0 = none, 2 = weak, 4 = moderate, 6 = strong, 8 = extremely strong). Samples were evaluated in triplicate; results presented as means in radar charts. The sensory training protocols targeting specific aroma attributes were developed following the experimental framework outlined in Liu et al. (2022).

### 2.4 Comprehensive two-dimensional gas chromatography-time-of-flight mass spectrometry (GC × GC-TOF-MS) analysis

#### 2.4.1 Sample preparation

Internal standard (ethyl decanoate) was diluted to 86.2 ppm in n-hexane. Powdered tea (1.5 g) was mixed with saturated NaCl (2 mL) and spiked with internal standard (2 μL). Automated SPME (PAL CTC system) was performed at 60 °C, 450 rpm (15 min equilibrium), followed by PDMS/CAR/DVB fiber extraction (30 min). Desorption occurred in the GC inlet (250 °C, 3 min; splitless mode). Triplicate analyses were conducted.

#### 2.4.2 GC × GC-TOF-MS instrumental conditions

GC × GC: Primary column: DB-WAX (30 m × 0.25 mm × 0.25 μm); secondary column: DB-17 MS (1.2 m × 0.18 mm × 0.18 μm); modulation period: 6.0 s. Inlet: 250 °C (splitless); carrier gas: He (>99.999%; 1 mL/min). Oven program: 40 °C (5 min) → 240 °C (4 °C/min; hold 5 min).

TOF-MS: EI source (70 eV); ion source: 230 °C; transfer line: 240 °C; detector: −1650 V; mass range: 47–350 amu; acquisition: 101 spectra/s.

### 2.5 Identification and quantification of volatile compounds

Chromatographic data were processed using Canvas software. Compounds were identified via NIST20 library matching (forward/reverse match >700/800; RI deviation <30). Concentrations were calculated by internal standard method:

$$C=\frac{A*C_{i}}{A_{i.}}$$

where: *C* = compound concentration (μg/L); *A* = compound peak area; *A*_*i*_ = internal standard peak area; *C*_*i*_ = internal standard concentration (μg/L).

### 2.6 Calculation of OAV and ACI for volatile compounds

Odor thresholds (OT) in water were referenced (Bao et al., 2025; Chen et al., 2024; Guo, Ho, Schwab, & Wan, 2021; Zhai et al., 2022). Odor activity value (OAV) and aroma contribution index (ACI) were calculated as:

$${\mathrm{OAV}}_{i}=\frac{C_{i}}{{\mathrm{OT}}_{i}}$$

where: *Ci* = concentration of the compound (μg/L); *OTi* = odor threshold of the compound in water (μg/L).

$$\mathrm{ACI}\left(\%\right)=\frac{{\mathrm{OAV}}_{i}}{{\mathrm{OAV}}_{T}}*100\%$$

where: *ACI* = aroma contribution index (%); *OAV*_*i*_ = OAV of the compound; *OAV*_*T*_ = total OAV of all volatile compounds.

### 2.7 Statistical analysis

Multivariate statistical analyses, including radar plots, heatmaps, principal component analysis (PCA), and orthogonal partial least squares-discriminant analysis (OPLS-DA), were performed using the Metware Cloud platform (https://cloud.metware.cn/). All experiments were performed with three independent replicates.

## 3 Results and discussion

### 3.1 Sensory evaluation reveals directed regulation of flavor profiles by processing techniques

**Fig. 2:**
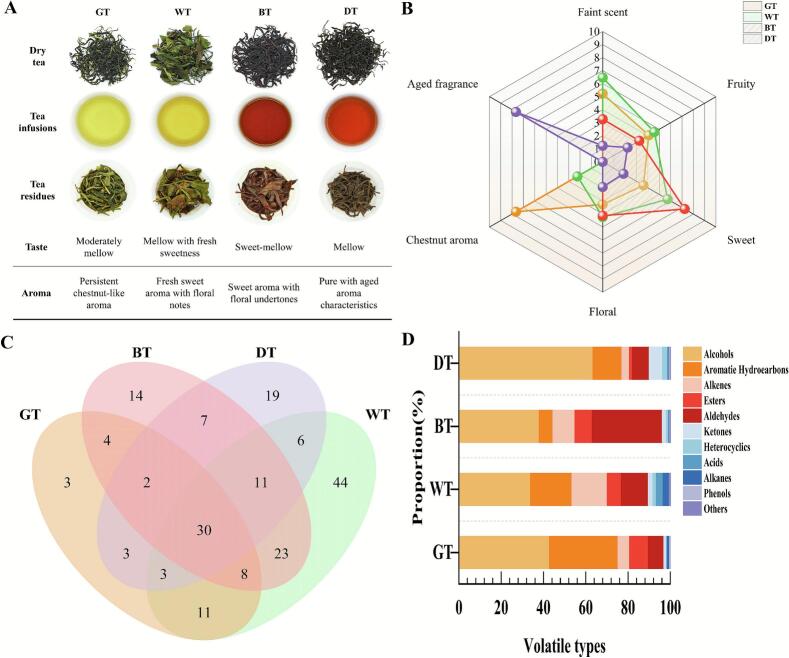
Sensory and aroma profiles of Fuding Dabai tea types . (A) sensory evaluation; (B) aroma intensity radar map; (C) venn diagram of aroma compounds; (D) aroma-type distribution. WT: white tea, GT: green tea, BT: black tea, DT: dark tea. (For interpretation of the references to colour in this figure legend, the reader is referred to the web version of this article.)

Appearance, liquor colour, and infused leaves exhibited significant differences across tea types (Fig. 2A). Quantitative sensory evaluation (Fig. 2B) based on six aroma attributes (fresh, fruity, sweet, floral, chestnut-like, and aged aroma) demonstrated GT's dominance in chestnut-like aroma (*p* < 0.05) with superior persistence. WT showed the highest intensity of fresh aroma intensity (23.71% > GT), accompanied by pronounced sweet and floral notes. BT exhibited enhanced sweet aroma (26.18% > WT), followed by floral character. DT displayed a predominant aged aroma contribution (>51.57%), distinguishing it from other types. These results confirm that processing reshapes the aroma foundation through modulation of the “enzyme inhibition-oxidation-fermentation” biochemical axis, establishing processing as the core driver of aroma differentiation (Xu et al., 2023).

### 3.2 Volatile compound profiles reveal process-specific characteristics

GC × GC-TOF MS analysis identified 187 volatile compounds, with counts varying significantly across tea types: 136 in WT, 99 in BT, 81 in DT, and 64 in GT (Table S1). The total content ranked as WT (2349.42 μg/L) > BT (1091.56 μg/L) > DT (954.21 μg/L) > GT (533.53 μg/L). Chemical class distributions (Fig. 2D) comprised alcohols (33.86–63.41%), aromatic hydrocarbons (6.49–32.50%), aldehydes(7.33–33.17%), alkenes (3.53–16.72%),esters (1.40–8.83%), ketones (1.24–6.28%), acids (0.31–3.21%), alkanes (0.20–2.97%), heterocyclics(0.37–2.38%), phenols (0.13–0.35%),and others (0.06–0.29%), with alcohols, ketones, and aldehydes predominating.

Venn diagram analysis (Fig. 2C) visually illustrates the compositional differences in volatile compounds among the four tea types. Statistical testing (*p* < 0.01, ANOVA) confirmed significant inter-group variation in volatile profiles. The diagram shows that only 30 volatile compounds were common to all four tea types, while GT, WT, BT, and DT contained 3, 14, 19, and 44 unique volatile compounds, respectively. Stacked bar plots further quantified these differences: alcohols dominated in WT (795.49 μg/L) and DT (605.06 μg/L), aromatic hydrocarbons were most enriched in WT (460.56 μg/L), and aldehydes were highly expressed in BT (362.05 μg/L) and WT (300.95 μg/L).

In GT, linalool (43.09 μg/L) and (*E*)-furanoid linalool oxide (29.30 μg/L) established a floral-fruity aroma foundation, while methyl salicylate (27.91 μg/L) functioned as a critical carrier of chestnut-like aroma (Wang, Ma, et al., 2020), its accumulation driven by glycoside pyrolysis during high-temperature fixation (280 °C). WT exhibited substantially elevated levels of methyl salicylate (114.06 μg/L; +308% vs. GT) and (*E*)-furanoid linalool oxide (102.65 μg/L; +250%), originating from the glycoside hydrolysis and lipoxygenase (LOX) pathway activated during prolonged withering (Bao et al., 2025). Concurrently, benzeneacetaldehyde (99.1 μg/L) contributed distinctive sweetness via Strecker degradation (Chen et al., 2019). BT demonstrated enhanced benzeneacetaldehyde (128.06 μg/L) and methyl salicylate (70.64 μg/L), derived from amino acid hydrolysis, which imparted floral-sweet notes. The characteristic sweet aroma of BT further arose from synergistic effects between the LOX pathway product (*E*)-2-hexenal (71.71 μg/L) and benzeneacetaldehyde (Wu et al., 2023). In DT, pile fermentation facilitated *β*-glucosidase-catalyzed generation of benzyl alcohol (69.85 μg/L) and (*E*)-furanoid linalool oxide (63.97 μg/L) by *Aspergillus niger* (Yan et al., 2024). These compounds masked grassy odors (e.g., (*E*)-2-hexenal) and synergized with α-terpineol (70.09 μg/L) to shape the aged aroma profile, aligning with established fermentation-derived aroma transformation mechanisms (Liu et al., 2022).

Furthermore, studies elucidating the biosynthetic mechanism behind white tea's distinctive aroma profile further establish glycoside hydrolysis as the core biosynthetic route for its characteristic volatile compounds. Specifically, glycosidically bound volatiles (GBVs), which act as aroma precursors, release characteristic aroma substances such as (*E*)-furanoid linalool oxide through the glycoside hydrolysis pathway during processing steps including withering (Chen et al., 2019). These compounds are not only essential components of white tea aroma but also their significantly elevated levels constitute the key factor that distinguishes the aroma characteristics of white tea from those of other tea types such as green tea.

### 3.3 Screening of key aroma compounds and analysis of contribution mechanisms

Characteristic tea aromas depend critically on key VOC concentrations and odor thresholds (Qin, Wang, et al., 2023). Odor activity value (OAV) screening identified 35 key compounds (OAV ≥ 1) (Table 1). High-OAV compounds (OAV > 100) defined aroma bases, while medium-low OAV compounds (1 ≤ OAV ≤ 100) enhanced complexity via synergistic effects, collectively constructing the perceptual model (Wang, Hu, et al., 2025). Key compound distributions were process-dependent: GT (13), WT (30), BT (21), DT (15), with significant cross-tea OAV variations.

**Table 1:** Key aroma-active compounds in different tea types processed from Fuding Dabai.

No	Compounds	Aroma description	Threshold ^a^(μg/L)	Odor Activity Value (OAV)				Aroma contribution index (ACI)(%)			
				GT	WT	BT	DT	GT	WT	BT	DT
1	1-Hexanol	Green, grassy	5.6	0.54	4.27	0.97	0.35	0.06	0.08	0.04	0.01
2	(*Z*)-3-Hexenol	Green	3.9	/	8.96	6.19	6.69	/	0.17	0.25	0.25
3	(*E*)-Linalool oxide (furanoid)	Floral, woody	2	14.65	51.33	/	31.99	1.53	0.98	/	1.21
4	1-Octen-3-ol	Green, oily	1	11.89	17.35	4.62	6.30	1.24	0.33	0.19	0.24
5	Linalool	Floral, sweet, woody	0.22	195.86	84.32	243.50	/	20.49	1.61	9.97	/
6	1-Octanol	Green, citrus, fatty, coconut-like	0.022	181.82	804.55	/	/	19.03	15.37	/	/
7	(*E*)-2-Octen-1-ol	Green	0.1	/	57.70	16.10	/	/	1.10	0.66	/
8	1-Nonanol	Fresh, green, floral, rose-like	0.0455	/	337.80	/	/	/	6.45	/	/
9	(*Z*)-3-Nonen-1-ol	Fresh, waxy, green, mushroom-like	0.209	/	34.40	/	/	/	0.66	/	/
10	(*E,Z*)-3,6-Nonadien-1-ol	Sweet, fresh, green, fruity	0.003	/	1440.00	/	/	/	27.51	/	/
11	Geraniol	Rose-like, sweet	3.2	2.95	3.50	16.57	6.02	0.31	0.07	0.68	0.23
12	Benzyl alcohol	Sweet, floral	3	3.85	29.97	16.97	23.28	0.40	0.57	0.69	0.88
13	Hexanal	Grassy, green, fresh, fatty	2.4	2.45	3.43	2.48	/	0.26	0.07	0.10	/
14	(*E*)-2-Hexenal	Green, leafy, fruity	0.08	/	380.13	896.38	/	/	7.26	36.70	/
15	Nonanal	Fatty, citrus, green	1.1	5.97	23.34	27.46	10.79	0.62	0.45	1.12	0.41
16	(*E,E*)-2,4-Heptadienal	Stale, fatty	0.032	/	149.06	155.31	1009.06	/	2.85	6.36	38.04
17	(*E,Z*)-2,6-Nonadienal	Cucumber-like	0.0045	/	/	/	957.78	/	/	/	36.11
18	*β*-Cyclocitral	Herbal, mint	3	0.38	2.76	1.14	2.17	0.04	0.05	0.05	0.08
19	Benzeneacetaldehyde	Honey-like	4	/	24.78	32.02	0.86	/	0.47	1.31	0.03
No	Compounds	Aroma description	Threshold ^a^(μg/L)	Odor Activity Value (OAV)				Aroma contribution index (ACI)(%)			
				GT	WT	BT	DT	GT	WT	BT	DT
20	Safranal	Woody, spicy, herbal	0.7	/	3.03	/	3.21	/	0.06	/	0.12
21	(*E*)-2-Decenal	Green, fatty	0.4	/	/	/	14.53	/	/	/	0.55
22	Heptanal	Fatty, green	0.033	389.09	/	65.45	/	40.71	/	2.68	/
23	(*E*)-2-Heptenal	Green, tallow-like	0.08	/	/	34.63	96.00	/	/	1.42	3.62
24	(*E*)-Citral	Lemon-like	0.1	/	/	117.10	/	/	/	4.79	/
25	2-Methyl-butanal	Pekoe scent, malt, sweet, fruity, cocoa	1.5	/	21.43	/	/	/	0.41	/	/
26	Geranylacetone	Fresh, rose-like, floral, green, fruity	0.4	/	5.63	/	/	/	0.11	/	/
27	(*E*)-*β*-Ionone	Violet-like, woody	0.007	108.57	1508.57	775.71	360.00	11.36	28.82	31.76	13.57
28	Jasmone	Floral, jasmine-like	0.7	2.57	6.93	9.73	1.36	0.27	0.13	0.40	0.05
29	6-Methyl-5-hepten-2-one	Citrus, fruity, apple-like	0.16	19.13	66.69	12.44	121.38	2.00	1.27	0.51	4.58
30	d-Limonene	Fruity, lemon-like	1.2	15.26	47.16	/	/	1.60	0.90	/	/
31	Terpinolene	Fresh, woody, sweet, piney, citrus	0.2	/	86.75	/	/	/	1.66	/	/
32	Myrcene	Woody, resinous, musty	15	/	6.34	3.41	0.36	/	0.12	0.14	0.01
33	Methyl salicylate	Wintergreen, peppermint	40	0.70	2.85	1.77	0.17	0.07	0.05	0.07	0.01
34	Pentanoic acid	Fruit	0.15	/	6.27	/	/	/	0.12	/	/
35	4-Hexanolide	Caramel, nutty, roasted, sweet	0.26	/	14.38	2.62	/	/	0.27	0.11	/

Note: ^a^All odor thresholds were obtained from references (Bao et al., 2025; Chen et al., 2024; Guo et al., 2021; Zhai et al., 2022); “/” means the missing OAV and ACI; Compounds are listed if their OAV ≥ 1 in at least one tea type (GT, WT, BT, or DT).

In GT, heptanal (OAV = 389.09) – a lipid peroxidation derivative imparting grassy/fruity nuances – synergistically formed the dominant chestnut-like aroma with linalool (OAV = 195.86), aligning with prior findings in chestnut-type green teas (Zhu et al., 2018). Synergistic effects exist between linalool and (*E*)-*β*-ionone as well as between hexanal and (*E*)-*β*-ionone, and these effects involve hydrophobic interactions and hydrogen bonding that alter the aroma perception of individual substances and thereby enhance the overall chestnut-like flavor characteristics (Zhu et al., 2025). White tea's (WT) signature fresh aroma was primarily driven by abundant LOX-pathway derivatives generated during withering: (*E*)-*β*-ionone (OAV = 1508.57) and (*E,Z*)-3,6-nonadien-1-ol (OAV = 1440.00) (Bao et al., 2025). Black tea's (BT) characteristic sweet aroma originated from odor interaction between (*E*)-2-hexenal (OAV = 896.38) and the process-specific compound (*E*)-citral (OAV = 117.10), with the latter's withering-dependent accumulation confirming established sweet-aroma formation mechanisms (Wu et al., 2023). Dark tea's (DT) aged aroma profile was principally shaped by (*E,E*)-2,4-heptadienal (OAV = 1009.06) and (*E,Z*)-2,6-nonadienal (OAV = 957.78), generated via microbial extracellular enzyme-catalyzed lipid oxidation during pile fermentation (Liu et al., 2022). Critically, although (*E,Z*)-2,6-nonadienal inherently contributes grassy notes, competitive olfactory receptor occupation by benzyl alcohol (OAV = 23.28) facilitated its sensory transformation into an aged-aroma component – a phenomenon consistent with established fermented-food aroma modulation patterns (Yan et al., 2024).

Medium-low OAV compounds (1 ≤ OAV ≤ 100) enhanced aroma characteristics through synergistic effects: in WT, 2-methyl-butanal (OAV = 21.43) and pentanoic acid (OAV = 6.27) as unique compounds imparted pekoe and fruity aromas through Strecker degradation (Lin et al., 2024); in BT, nonanal (OAV = 27.46) showed significant positive correlation with honey-like sweetness (Qin, Zhou, et al., 2023). Shared compounds also showed process specificity: benzyl alcohol had the highest OAV in WT (29.97), originating from lipid oxidation activated during withering; 6-methyl-5-hepten-2-one reached an OAV of 121.38 in DT, identified as an aged aroma marker (Zhang et al., 2021).

To further quantify compound contribution intensity, the aroma contribution index (ACI > 1%) was introduced to screen core compounds: in GT, heptanal (ACI = 40.71%) and linalool formed the chestnut-like aroma skeleton; WT's unique 1-nonanol (OAV = 337.80, ACI = 6.45%) drove the fresh aroma; in BT, (*E*)-citral (ACI = 4.79%) shaped the sweet-fruity aroma; in DT, (*E,Z*)-2,6-nonadienal (ACI = 36.11%) served as the aged aroma carrier. These findings reveal three aroma regulation pathways: enzymatic (withering-activated LOX pathway→C6/C9 aldehyde/alcohol accumulation), thermochemical (fixation locking heat-stable compounds), and microbial (pile-fermentation inducing masking effects→transformation from grassy to aged notes) (Ming et al., 2025; Zhai et al., 2022; Zhang et al., 2021). Within the studied framework, the white tea (WT) process yielded the highest number of key aroma compounds (*n* = 30) and the greatest total odor activity value (ΣOAV = 5233.65). This outcome suggests that the minimal processing involved in white tea manufacture, which is characterized by prolonged withering without fixation, more effectively retains and transforms the cultivar's inherent precursors into potent floral and fruity volatiles (e.g., (*E*)-*β*-ionone), thereby maximizing its distinctive aroma potential.

### 3.4 Multivariate statistics reveal aroma differentiation patterns and sensory correlation mechanisms

#### 3.4.1 PCA analysis of aroma compound patterns in diverse tea types

**Fig. 3:**
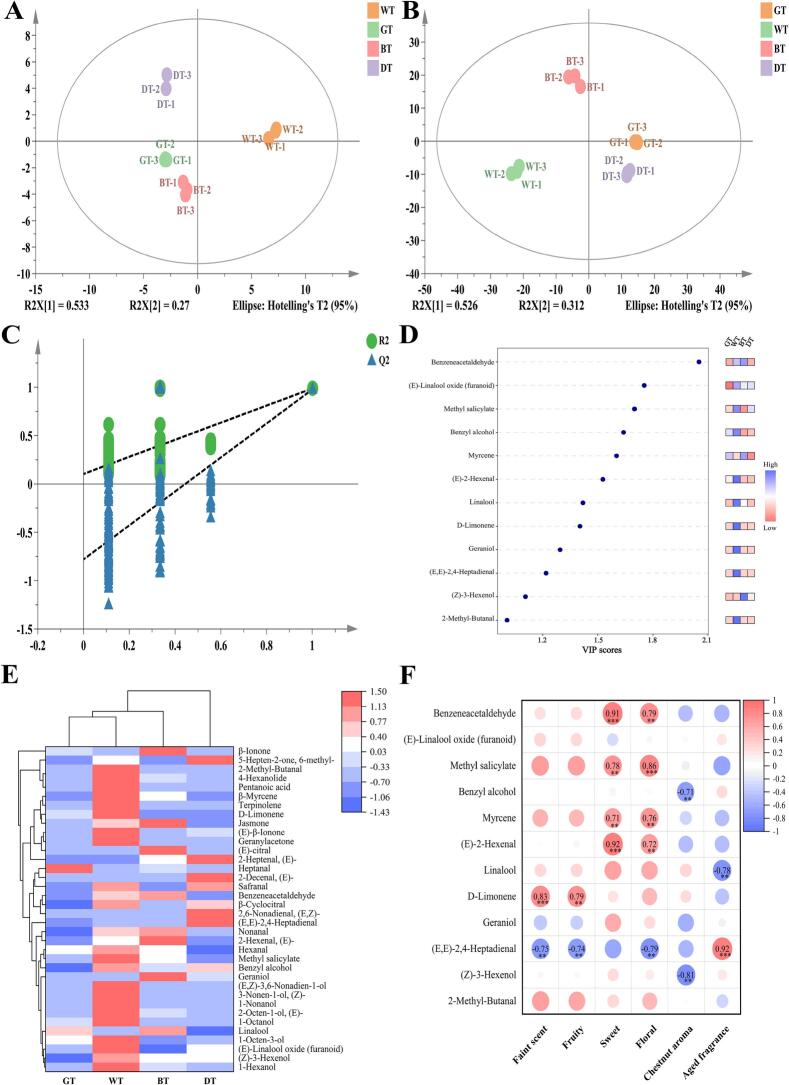
Multivariate statistical analysis of volatile compounds across tea types. (A) PCA score plot; (B) OPLS-DA score plot; (C) permutation test; (D) VIP screening (VIP > 1, *p* < 0.05); (E) hierarchical clustering analysis; (F) correlation heatmap. WT: white tea, GT: green tea, BT: black tea, DT: dark tea, PCA: principal component analysis, OPLS-DA: orthogonal partial least squares discriminant analysis, VIP: variable importance in projection. (For interpretation of the references to colour in this figure legend, the reader is referred to the web version of this article.)

Principal component analysis (PCA) of 35 key aroma compounds with OAV > 1 showed PC1 (53.3%) and PC2 (27.0%) cumulatively explained 80.3% variance (Fig. 3A). PC1 separated fermented teas (BT/DT) from lightly fermented teas (WT), with loading positively correlated with oxidation degree (|*r*| > 0.85); PC2 distinguished lightly processed teas (GT/WT) from deeply fermented tea (DT), associated with lipid oxidation products. Good within-group repeatability (RSD < 12%) confirmed processing differences as the core driver of aroma differentiation. The clear separation along PC1 highlights the fundamental impact of fermentation on the volatile profile, with BT and DT characterized by higher levels of microbial-derived compounds and enzymatic oxidation degradation products.

#### 3.4.2 OPLS-DA for discriminating key aroma compounds in various tea types

To further explore differences among four tea types processed by different methods, orthogonal partial least squares-discriminant analysis (OPLS-DA) models were constructed based on PCA results. The model (R^2^X = 0.526, Q^2^ = 0.312) was validated by permutation test (R^2^ intercept = 0.104, Q^2^ intercept = −0.78), screening 12 differentiation markers with VIP > 1 (Fig. 3B-D). Alcohols (5 compounds, e.g., (*E*)-furanoid linalool oxide VIP = 1.75, benzyl alcohol VIP = 1.64) and aldehydes (4 compounds, e.g., benzeneacetaldehyde VIP = 2.05) contributed 75% cumulatively, indicating these two classes as the primary contributors to tea aroma differentiation (Zhang et al., 2019). The high VIP values of benzeneacetaldehyde and linalool oxide across multiple tea types highlight their fundamental role in tea aroma, despite varying contributions to different sensory profiles (Liu et al., 2022; Zhai et al., 2022).

#### 3.4.3 HCA for discriminating aroma profiles across different tea categories

Hierarchical clustering (HCA) further verified this conclusion (Fig. 3E): GT/WT/BT clustered together, while DT formed a separate cluster due to pile-fermentation-derived unsaturated aldehydes (e.g., (*E,E*)-2,4-heptadienal). Compound expression patterns showed significant process characteristics: high expression of heptanal and linalool in GT reflected fixation locking lipid oxidation products; WT enriched C6/C9 alcohols (*E,Z*)-3,6-nonadien-1-ol, hexanol, 1-octen-3-ol etc.) and terpinolene from withering-activated LOX pathway; BT accumulated terpenoid alcohols (linalool, geraniol), benzeneacetaldehyde,and (*E*)-citral, confirming fermentation-promoted glycoside hydrolysis; DT was dominated by lipid oxidation products like (*E,E*)-2,4-heptadienal, (*E,Z*)-2,6-nonadienal, and (*E*)-2-decenal. The unique clustering of DT underscores the transformative effect of microbial pile fermentation on the volatile profile, generating distinct aged aroma compounds not found in other tea types (Liu et al., 2022).

#### 3.4.4 Heatmap correlation analysis constructs compound-sensory interaction networks

Heatmap analysis (Fig. 3F) systematically revealed quantitative relationships between key aroma compounds and sensory attributes: floral and sweet attributes showed highly significant positive correlations with methyl salicylate (*r* = 0.86, *p* < 0.001) and benzeneacetaldehyde(*r* = 0.91, *p* < 0.001), whose high expression in lightly processed teas (WT withering) confirmed their core roles as fresh/sweet aroma base; aged aroma showed strong positive correlation with (*E,E*)-2,4-heptadienal (*r* = 0.92, *p* < 0.001), identified as a marker product of linolenic acid *β*-oxidation catalyzed by *Aspergillus niger* during pile fermentation (Liu et al., 2022). Negative effect mechanisms included: chestnut-like aroma negatively correlated with benzyl alcohol (*r* = −0.71, *p* < 0.01) due to phenolic precursor degradation during fermentation; fresh-sweet aroma negatively correlated with (*E,E*)-2,4-heptadienal (*r* = −0.69, *p* < 0.05), reflecting aroma loss from heat-induced isomerization (Yan et al., 2024).

Key findings revealed process-directed regulation of compound functions: linalool and d-limonene synergistically shaped GT's chestnut-like aroma through floral-citrus notes (Zou, Tang, Yang, Guo, & Song, 2023), but showed significant negative correlation with aged aroma in DT (*r* = −0.78, *p* < 0.01; *r* = −0.62, *p* < 0.05), indicating sensory function reprogramming of the same compound under different processes — non-fermentation processes retain pleasant floral-fruity notes while pile-fermentation triggers transformation to woody notes; (*E*)-2-hexenal positively correlated with sweet aroma in BT (*r* = 0.92, *p* < 0.001) but showed no significant correlation in DT, confirming pile fermentation microbes eliminate undesirable grassy notes through *β*-oxidation pathways (Wu et al., 2023), highlighting microbial metabolism's ability to transform off-flavor compounds; benzeneacetaldehyde as a cross-tea core positive regulator (*r* > 0.80 in WT/BT), whose accumulation is dually regulated by withering duration and fermentation intensity (Chen et al., 2019), demonstrates the universality of amino acid Strecker degradation pathways across different processes. These correlations underscore the complex interplay between volatile compounds and sensory perception, where the flavor expression of compounds is highly context-dependent on the chemical basis established during processing.

### 3.5 Molecular aroma wheel characterization of characteristic aroma profiles in various types of Fuding Dabai tea

**Fig. 4:**
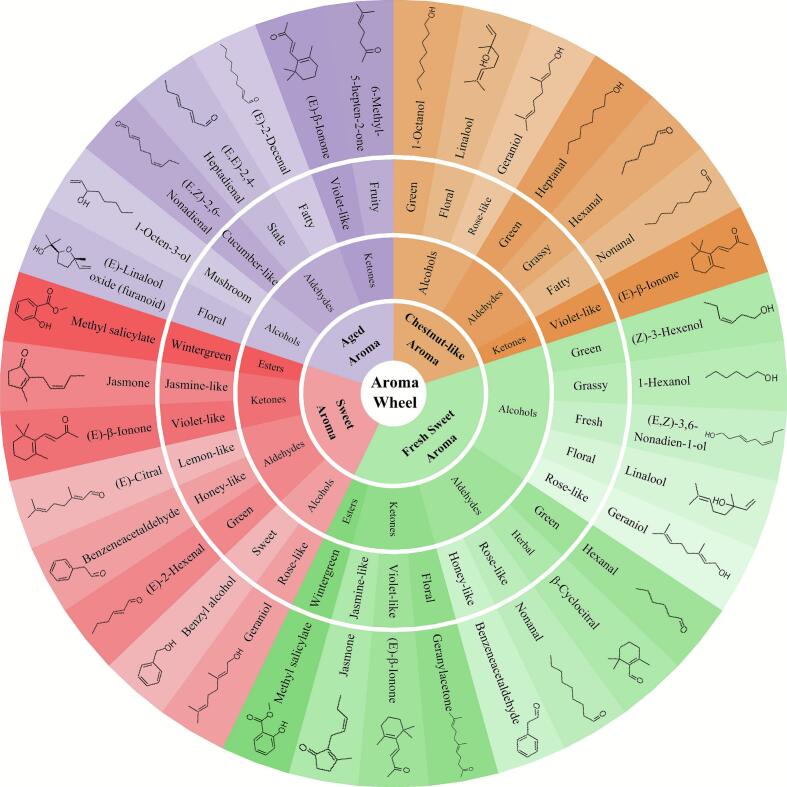
Molecular aroma wheel characterizing key odorants in different tea types.

In order to systematically elucidate the characteristic aroma composition of different categories of Fuding Dabai tea, this study classified key aromatic compounds according to their sensory attributes and grouped them based on olfactive similarities, thereby constructing a molecular aroma wheel for the four principal tea types derived from this cultivar (Fig. 4). This wheel delineates aroma characteristics into four fundamental categories, each further subdivided into specific aromatic typologies.

Green tea (chestnut-like aroma): the representative aroma profile of green tea is dominated by alcohols, aldehydes, and ketones. Notable examples include linalool (imparting floral and sweet notes), 1-octanol (conveying a green-citrus character), nonanal (exhibiting a fatty-citrus aroma), heptanal (providing a fatty-green accent), and (*E*)-*β*-ionone (contributing a violet-woody aroma). The OAVs of these constituents all exceed 1, indicating their significant contribution to the aroma profile of green tea, particularly the chestnut-type aroma (Zhu et al., 2018; Wang, Hua, et al., 2020). These compounds operate not only individually but also synergistically, accentuating the dominant fruity and nutty nuances characteristic of this tea type.

White tea (delicate floral scent): the aromatic essence of white tea is principally constituted by alcohols, aldehydes, ketones, and esters. Key components comprise linalool (floral), (*E,Z*)-3,6-nonadien-1-ol (sweet, fresh, green aroma), 1-hexanol (green grassy note), (*Z*)-3-hexenol (fresh green scent), hexanal (green character), benzeneacetaldehyde (honey-like), *β*-cyclocitral (herbaceous quality), (*E*)-*β*-ionone (violet-woody note), geranyl acetone (floral-fruity tone), and methyl salicylate (wintergreen-minty aroma) (Wang, Ma, et al., 2020; Bao et al., 2025). These compounds collectively underpin the refined, layered, and subtly complex floral bouquet emblematic of white tea.

Black tea (sweet aroma): the aromatic architecture of black tea incorporates alcohols, aldehydes, ketones, and terpenes. Representative compounds include geraniol (rosy sweet aroma), benzyl alcohol (sweet floral note), (*E*)-2-hexenal (green fruity character), benzeneacetaldehyde (honey-like), (*E*)-citral (lemon-like freshness), (*E*)-*β*-ionone (violet-woody nuance), jasmone (jasmine sweet scent), and methyl salicylate (wintergreen mint) (Wang et al., 2024; Wu et al., 2023). These volatiles are extensively generated and transformed during the withering, rolling, and fermentation stages of black tea processing. Their synergistic interplay culminates in the characteristic sweet, floral, and fruity flavor paradigm defining black tea.

Dark tea (aged aroma): the distinctive aroma of dark tea is largely attributable to alcohols, aldehydes, and ketones. Major constituents encompass (*E*)-linalool oxide (floral-woody scent), (*E,E*)-2,4-heptadienal (stale note), (*E,Z*)-2,6-nonadienal (cucumber-green aroma), (*E*)-*β*-ionone (violet-woody character), and 6-methyl-5-hepten-2-one (green-fruity nuance) (Liu et al., 2022; Zhang et al., 2021). These compounds predominantly originate from oxidative, degradative, and aging reactions during the post-fermentation process, collectively conferring the profound, mellow, and aged organoleptic properties characteristic of dark tea.

Expanding upon the conventional sensory flavor wheel, this investigation innovatively introduces a molecular aroma wheel model, thereby establishing a precise visual correspondence between sensory descriptors and aroma-active molecules. The findings enhance the comprehension of the molecular aromatic signatures inherent to different Fuding Dabai tea types, facilitating a systematic integration of chemical data with sensory attributes. This approach permits a more scientifically rigorous deciphering of its “aroma code.” By categorizing aroma properties according to molecular structures, this research not only lays a molecular foundation for the objective quantitative assessment of aroma quality across various Fuding Dabai tea types but also affords a novel methodological framework for tea flavor studies. Furthermore, this model holds potential for guiding future applications in process optimization, quality control, and product traceability.

### 3.6 Elucidation of aroma formation pathways regulated by processing

**Fig. 5:**
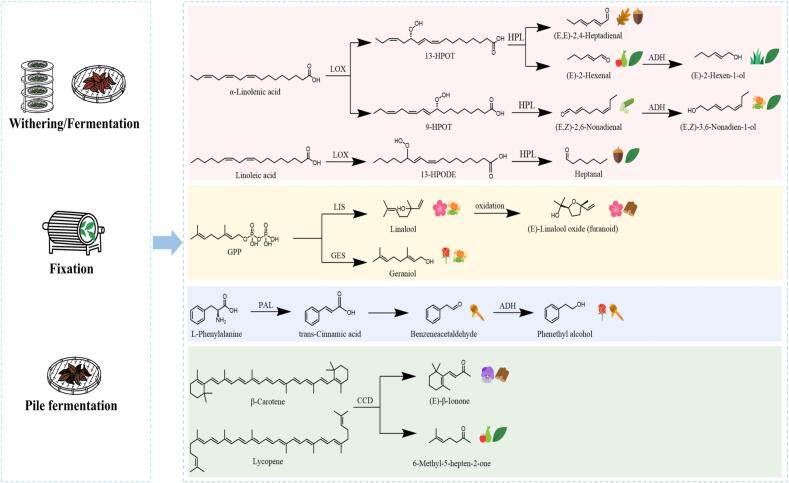
Proposed biosynthetic pathways of key aroma compounds during tea processing. LOX: lipooxygenase, HPL: hydroperoxide lyase, ADH: alcohol dehydrogenase, LIS: linalool synthase, GES: geraniol synthase, GPP: geranyl diphosphate, PAL: phenylalanine ammonia-lyase, CCD: carotenoid cleavage dioxygenase.

The divergent aroma profiles observed across the four tea types can be attributed to the activation of distinct biochemical pathways during processing, with three primary regulation mechanisms identified (Fig. 5). In white tea processing, prolonged withering activates lipoxygenase (LOX) and glycosidase enzymes, leading to the accumulation of C6/C9 aldehydes and alcohols such as (*E,Z*)-3,6-nonadien-1-ol through lipid oxidation and glycoside hydrolysis. Conversely, high-temperature fixation during green tea processing inhibits enzymatic activity while promoting thermal degradation of precursors—exemplified by the pyrolysis of glycosides into linalool—thereby preserving fresh and chestnut-like notes. For dark tea, pile fermentation facilitates microbial-catalyzed *β*-oxidation of lipids by microorganisms such as *Aspergillus niger*, generating unsaturated aldehydes including (*E,E*)-2,4-heptadienal and transforming grassy notes into the characteristic aged aroma. These pathway activations are directly influenced by key processing parameters—such as withering duration, fermentation temperature, and thermal treatment intensity—providing a coherent biochemical basis for the observed aroma differentiation among tea types.

## 4 Conclusion

This study demonstrates that processing technology serves as the primary determinant of aroma differentiation in Fuding Dabai tea, with fermentation degree accounting for over 50% of the variation in volatile profiles (PC1: 53.3% variance). The aroma regulation follows three distinct pathways: enzymatic (withering-activated LOX pathway, generating compounds such as (*E*)-*β*-ionone with OAV = 1508.57), thermochemical (fixation-derived products like heptanal with OAV = 389.09), and microbial (pile fermentation producing aged-aroma markers including (*E,E*)-2,4-heptadienal with OAV = 1009.06). These findings provide actionable insights for aroma-oriented manufacturing: white tea processing best exploits the cultivar's potential (30 key aroma compounds, total OAV = 5233.65), while targeted microbial fermentation enables directional enhancement of aged aroma in dark tea. The molecular aroma wheel model further establishes a direct link between sensory attributes and chemical composition, offering a systematic framework for quality control and process optimization. We note that this study focused on a single cultivar under controlled conditions. Future work should explore commercial-scale validation and multi-variety comparisons to extend the applicability of these aroma regulation principles. The integration of such molecular insights with intelligent manufacturing systems will pave the way for precision tea processing and customized flavor design.

## CRediT authorship contribution statement

**Panpan Liu:** Writing – review & editing, Writing – original draft, Resources, Methodology, Investigation, Funding acquisition, Formal analysis, Data curation, Conceptualization. **Jia Chen:** Writing – original draft, Formal analysis, Data curation. **Lin Feng:** Writing – original draft, Investigation. **Shiwei Gao:** Investigation, Funding acquisition. **Shengpeng Wang:** Formal analysis, Data curation. **Jinjin Xue:** Methodology, Investigation. **Xueping Wang:** Formal analysis. **Fei Ye:** Formal analysis. **Anhui Gui:** Formal analysis. **Zhi Yu:** Writing – review & editing, Writing – original draft, Methodology. **Pengcheng Zheng:** Writing – review & editing, Writing – original draft, Resources.

## Ethics statement

This study involving human sensory evaluation was conducted in accordance with the ethical principles of the Declaration of Helsinki. Institutional ethical approval for such sensory studies was not required. All procedures complied with relevant national operational standards. Prior to participation, all panelists provided written informed consent using a “Sensory Panelist Consent Form and Statement.” We implemented protocols to protect participants' rights and privacy, including voluntary participation, full disclosure of study requirements and risks, protection of confidential data, and the right to withdraw at any time without reason.

## Declaration of competing interest

The authors declare that they have no known competing financial interests or personal relationships that could have appeared to influence the work reported in this paper.

## Data Availability

Data will be made available on request.

## Appendix A Supplementary data

Supplementary material Image 1
