# Supplementary material for: Processing technology as aroma architect: OAV fingerprints decode differentiation and compatibility of key odorants in Fuding Dabai tea via GC×GC-TOF-MS and sensomics
Source: Food Chem X. 2026 Jan 13;34:103530. doi: 10.1016/j.fochx.2026.103530 (PMC12860358; doi:10.1016/j.fochx.2026.103530)
Supplement: Supplementary file 1 — Supplementary material [file mmc1.docx]

**Processing Technology as Aroma Architect: OAV Fingerprints Decode Differentiation and Compatibility of Key Odorants in** **Fuding Dabai tea via GC×GC-TOF-MS and Sensomics**

Panpan Liu^a,1^, Jia Chen^a,b,1^, Lin Feng^a^, Shiwei Gao^a^, Shengpeng Wang^a^, Jinjin Xue^a^, Xueping Wang^a^, Fei Ye^a^, Anhui Gui^a^, Zhi Yu^b,^*, Pengcheng Zheng ^a,^*

^a^ Key Laboratory of Tea Resources Comprehensive Utilization, Ministry of Agriculture and Rural Affairs, Hubei Qingzhuan Tea Engineering Research Centre, Fruit and Tea Research Institute, Hubei Academy of Agricultural Sciences, Wuhan, Hubei, 430064, China

^b^ College of Horticulture & Forestry Sciences, Huazhong Agricultural University, Wuhan, Hubei, 430070, China

*Corresponding Author

Email addresses: liuppitea@163.com (P. Liu), yuzhipl@163.com (Z. Yu), [zpct@hbaas.com](mailto:zpct@hbaas.com) (P. Zheng)

^1^ These authors contributed equally to this work

**Table S1**

The volatiles identified in four different tea types processed from Fuding Dabai.

| No | Name | CAS | Compounds | Aroma description | Contents（μg/L） | | | |
| --- | --- | --- | --- | --- | --- | --- | --- | --- |
|  |  |  |  |  | GT | WT | BT | DT |
| 1 | 3-Pentanol | 584-02-1 | Alcohols | Sweet, herbal, oily, nutty flavor，herbal | ND | 0.71±0.06 | ND | ND |
| 2 | 1-Penten-3-ol | 616-25-1 | Alcohols | Ethereal，green，tropical fruity | ND | 13.57±2.02 | 4.62±0.61 | ND |
| 3 | 3-Methyl-1-butanol | 123-51-3 | Alcohols | Burnt, cocoa-like, winey | ND | 5.86±1.38 | ND | ND |
| 4 | 1-Pentanol | 71-41-0 | Alcohols | Fermented | 9.27±0.75 | 7.39±1.95 | 1.64±0.55 | 10.26±1.73 |
| 5 | (*E*)-2-Penten-1-ol | 1576-96-1 | Alcohols | Mushroom | ND | 1.19±0.21 | 1.27±0.32 | ND |
| 6 | (*Z*)-2-Penten-1-ol | 1576-95-0 | Alcohols | Green, phenolic, medicinal, cherry,fruity | 0.76±0.21 | 22.42±2.08 | 16.27±2.09 | 2.23±0.24 |
| 7 | 1-Hexanol | 111-27-3 | Alcohols | Green, grassy | 3±0.81 | 23.91±3.91 | 5.43±1.27 | 1.98±0.61 |
| 8 | (*E*)-3-Hexenol | 928-97-2 | Alcohols | Green, leafy, grassy | ND | 4.22±0.59 | ND | ND |
| 9 | (*Z*)-3-Hexenol | 928-96-1 | Alcohols | Green | ND | 34.93±4.27 | 24.15±1.49 | 26.1±2.1 |
| 10 | (*E*)-2-Hexen-1-ol | 928-95-0 | Alcohols | Herbal, green | 0.45±0.09 | 22.13±2.01 | 13.33±2.88 | 0.73±0.21 |
| 11 | (*Z*)-2-Hexen-1-ol | 928-94-9 | Alcohols | Green | ND | 2.21±0.45 | ND | ND |
| 12 | (*E*)-Linalool oxide (furanoid) | 34995-77-2 | Alcohols | Floral, woody | 29.3±2.21 | 102.65±13.97 | ND | 63.97±4.56 |
| 13 | 1-Octen-3-ol | 3391-86-4 | Alcohols | Green, oily | 11.89±0.15 | 17.35±1.78 | 4.62±1.12 | 6.3±0.69 |
| 14 | 1-Heptanol | 111-70-6 | Alcohols | Green, sweet, leafy | ND | 11.7±1.97 | 1.42±0.37 | ND |
| 15 | 6-Methyl-5-hepten-2-one | 1569-60-4 | Alcohols | Citrus, fruity, apple-like | ND | 8.24±2.73 | 0.54±0.1 | 3.33±1.22 |
| 16 | Linalool oxide I | 5989-33-3 | Alcohols | Floral，woody | 20.42±0.69 | ND | ND | 137.91±11.45 |
| 17 | 2-Ethylhexanol | 104-76-7 | Alcohols | Sweet，citrus | 7.33±1.73 | 175.34±21.44 | 4.8±0.6 | 9.98±0.63 |
| 18 | Linalool | 78-70-6 | Alcohols | Floral, sweet, woody | 43.09±3.45 | 18.55±2.88 | 53.57±8.25 | ND |
| 19 | 1-Octanol | 111-87-5 | Alcohols | Green, citrus, fatty,  coconut-like | 4±0.61 | 17.7±3.3 | ND | ND |
| 20 | Terpinen-4-ol | 562-74-3 | Alcohols | Spicy, woody, earthy, citrus | ND | 0.86±0.41 | ND | 2.88±0.86 |
| 21 | 3,7-dimethylocta-1,5,7-trien-3-ol | 29957-43-5 | Alcohols | Fresh, floral, fruity | 13.43±1.66 | 18.85±2.14 | 15.11±1.62 | 20.96±2.69 |
| 22 | (*E*)-2-Octen-1-ol | 18409-17-1 | Alcohols | Green | ND | 5.77±0.51 | 1.61±0.17 | ND |
| 23 | 1-Nonanol | 143-08-8 | Alcohols | Fresh, fatty, floral，rose-like | ND | 15.37±0.51 | ND | ND |
| 24 | (*Z*)-3-Nonen-1-ol | 10340-23-5 | Alcohols | Fresh, waxy, green,mushroom-like | ND | 7.19±2.25 | ND | ND |
| 25 | (*E*)-3-Nonen-1-ol | 10339-61-4 | Alcohols | Mushroom-like | ND | ND | 3.25±1.07 | ND |
| 26 | *α*-Terpineol | 98-55-5 | Alcohols | Pleasant, floral | ND | 21.46±1.15 | 15.94±3.04 | 70.09±6.36 |
| 27 | Linalool oxide III | 14049-11-7 | Alcohols | Green,lemon-like | 14.08±1.64 | 43.39±2.32 | 37.25±5.32 | 39.84±4.2 |
| 28 | (*E,Z*)-3,6-Nonadien-1-ol | 56805-23-3 | Alcohols | Sweet, fresh, green,waxy, melon, fruity | ND | 4.32±1 | ND | ND |
| 29 | Linalool oxide IV | 39028-58-5 | Alcohols | waxy, melon, fruity | 25.64±0.6 | 64.44±6.75 | 45.87±5.11 | 50.6±5.17 |
| 30 | 7-Methyl-3-methylene-6-octen-1-ol | 13066-51-8 | Alcohols | - | ND | 2.83±0.55 | 1.55±0.15 | ND |
| 31 | Nerol | 106-25-2 | Alcohols | Fresh,floral, green, sweet | ND | 11.03±1.16 | 4.43±0.47 | 5.31±0.5 |
| 32 | (*Z*)-3,7-Dimethyl-3,6-octadien-1-ol | 5944-20-7 | Alcohols | Sweet rose fragrance, floral | ND | 6.91±2.35 | 2±0.45 | ND |
| 33 | Geraniol | 106-24-1 | Alcohols | Rose-like, sweet | 9.44±0.92 | 11.2±1.15 | 53.03±4.89 | 19.25±1.72 |
| 34 | Benzyl alcohol | 100-51-6 | Alcohols | Sweet, floral | 11.54±1.59 | 89.9±2.71 | 50.92±5.59 | 69.85±7.41 |
| 35 | (*E*)-2,6-Dimethyl-3,7-octadiene-2,6-diol | 13741-21-4 | Alcohols | - | ND | 1.37±0.28 | 1.63±0.09 | ND |
| 36 | Phenylethyl Alcohol | 60-12-8 | Alcohols | Floral, rose-like | 24.94±3.84 | ND | 50.18±2.03 | 63.49±4.21 |
| 37 | *p*-Cymen-7-ol | 536-60-7 | Alcohols | Pungent cumin scent, | ND | 0.53±0.05 | 0.49±0.16 | ND |
| 38 | Toluene | 108-88-3 | Aromatic Hydrocarbons | Sweet, aromatic | 14.46±1.21 | 6.79±0.32 | 3.51±1.07 | 8.88±1.03 |
| 39 | Ethylbenzene | 100-41-4 | Aromatic Hydrocarbons | Aromatic | 10.23±1.51 | 3.91±0.2 | ND | 2.95±0.21 |
| 40 | *o*-Xylene | 95-47-6 | Aromatic Hydrocarbons | Benzene-like peculiar fragrance | 10.26±0.82 | 12.59±1.37 | ND | 9.23±0.96 |
| 41 | *m*-Xylene | 108-38-3 | Aromatic Hydrocarbons | Fragrant | 14.44±2 | 29.17±3.27 | 2.12±0.49 | ND |
| 42 | *p*-Xylene | 106-42-3 | Aromatic Hydrocarbons | Plastic, green, pungent | 32.13±0.99 | 12.52±1.52 | 5.41±0.78 | 35.26±4.73 |
| 43 | Mesitylene | 108-67-8 | Aromatic Hydrocarbons | Aromatic, slightly sweet, gasoline-like or solvent-like odor | 2.83±0.89 | 24.07±5.21 | 3.95±1.25 | 4.54±0.99 |
| 44 | Styrene | 100-42-5 | Aromatic Hydrocarbons | Floral，balsamic | ND | 78.94±9.48 | ND | 31.87±0.72 |
| 45 | [4-Ethyltoluene](https://www.chemsrc.com/en/cas/622-96-8_960224.html) | 622-96-8 | Aromatic Hydrocarbons | - | 8.32±3.92 | 7.52±1.91 | 1.64±0.46 | 11.14±1.07 |
| 46 | 2-Ethyltoluene | 611-14-3 | Aromatic Hydrocarbons | - | ND | 16.73±2.92 | 3.32±0.34 | ND |
| 47 | 1,2,3-Trimethylbenzene | 526-73-8 | Aromatic Hydrocarbons | Plastic-like | 11.36±1.33 | 12.66±1.75 | ND | ND |
| 48 | Benzene, 1-methyl-3-propyl- | 1074-43-7 | Aromatic Hydrocarbons | - | 7.66±0.66 | 29.08±8.32 | 2.97±0.43 | ND |
| 49 | Butylbenzene | 104-51-8 | Aromatic Hydrocarbons | - | ND | 10.04±2.7 | ND | ND |
| 50 | Benzene, 4-ethyl-1,2-dimethyl- | 934-80-5 | Aromatic Hydrocarbons | - | ND | 12.4±1.02 | ND | ND |
| 51 | *α*-Methylstyrene | 98-83-9 | Aromatic Hydrocarbons | - | 3.61±0.27 | 9.02±0.86 | 1.55±0.41 | 1.85±0.13 |
| 52 | Benzene, (1-methylpropyl)- | 135-98-8 | Aromatic Hydrocarbons | - | ND | 13.72±2.47 | ND | ND |
| 53 | Benzene, 1,2,4-trimethyl- | 95-63-6 | Aromatic Hydrocarbons | Plastic-like | 14.9±2.09 | 31.74±1.91 | 24.82±1.63 | 3.72±0.45 |
| 54 | *m*-Cymol | 535-77-3 | Aromatic Hydrocarbons | - | 1.4±0.37 | 4.7±1.85 | ND | ND |
| 55 | 1H-Indene, 1-hexadecyl-2,3-dihydro- | 55334-29-7 | Aromatic Hydrocarbons | - | 1.44±0.18 | ND | 2.6±0.39 | 2.99±0.69 |
| 56 | Benzene, 1-ethyl-2,4-dimethyl- | 874-41-9 | Aromatic Hydrocarbons | - | 4.56±0.45 | 10.77±0.87 | ND | ND |
| 57 | Benzene, 1-ethenyl-3-methyl- | 100-80-1 | Aromatic Hydrocarbons | - | ND | 17.53±1.97 | ND | ND |
| 58 | Indane | 496-11-7 | Aromatic Hydrocarbons | - | 4.92±0.62 | 12.65±2.21 | ND | ND |
| 59 | *p*-Cymene | 99-87-6 | Aromatic Hydrocarbons | Mild and pleasant，terpenic | 5.26±0.94 | 8.45±1.49 | ND | ND |
| 60 | [*β*-Methylstyrene](https://www.chemsrc.com/en/cas/637-50-3_1114515.html) | 637-50-3 | Aromatic Hydrocarbons | - | ND | ND | ND | 2.77±0.21 |
| 61 | *o*-Cymene | 527-84-4 | Aromatic Hydrocarbons | Aromatic | ND | 4.8±0.97 | ND | ND |
| 62 | [*n*-Amylbenzene](https://www.chemsrc.com/en/cas/538-68-1_346914.html) | 538-68-1 | Aromatic Hydrocarbons | - | ND | 3.13±1.29 | ND | ND |
| 63 | Benzene, 1,2,4,5-tetramethyl- | 95-93-2 | Aromatic Hydrocarbons | Sour-sweet odor | 5.28±1.51 | 9.03±1.18 | ND | ND |
| 64 | Benzene, 1,2,3,4-tetramethyl- | 488-23-3 | Aromatic Hydrocarbons | - | 1.53±0.29 | 4.13±0.97 | 1.39±0.15 | ND |
| 65 | Benzene, 1-methyl-4-(1-methylethenyl)- | 1195-32-0 | Aromatic Hydrocarbons | Spicy, phenolic, clove-like, musty | 1.18±0.11 | 5.13±1.45 | 3.64±0.79 | 1.15±0.22 |
| 66 | Benzene, 1-methyl-4-butyl | 1595-05-7 | Aromatic Hydrocarbons | - | ND | 4.36±0.49 | ND | ND |
| 67 | Naphthalene, 1,2,3,4-tetrahydro- | 119-64-2 | Aromatic Hydrocarbons | Pungent menthol | 2.95±0.05 | 1.43±0.37 | 0.71±0.11 | 0.96±0.14 |
| 68 | Benzene, (3-octylundecyl)- | 5637-96-7 | Aromatic Hydrocarbons | - | ND | 13.13±3.14 | ND | ND |
| 69 | Naphthalene, 1,2,3,4-tetrahydro-2-methyl- | 3877-19-8 | Aromatic Hydrocarbons | - | 0.55±0.07 | 2.72±0.52 | ND | ND |
| 70 | Naphthalene | 91-20-3 | Aromatic Hydrocarbons | Pungent, tarry-like | 11.77±1.4 | 39.56±6.08 | 10.68±0.53 | 12.07±0.87 |
| 71 | (*E*)-Calamenene | 73209-42-4 | Aromatic Hydrocarbons | - | ND | 0.71±0.18 | 1.12±0.07 | ND |
| 72 | 1-Methylnaphthalene | 90-12-0 | Aromatic Hydrocarbons | Naphthyl, chemical | 2.35±0.04 | 7.43±2.72 | 1.29±0.45 | 1.26±0.19 |
| 73 | *n*-Caproic acid vinyl ester | 3050-69-9 | Esters | - | ND | 5.15±1.78 | ND | ND |
| 74 | Ethyl heptanoate | 106-30-9 | Esters | Fruity cognac and rum blend aroma，fruity | 5.19±1.4 | 11.14±2.62 | ND | ND |
| 75 | 2-Hexenoic acid, ethyl ester | 1552-67-6 | Esters | Fruity cognac and rum blend aroma，green，fruity | ND | 1.18±0.35 | ND | ND |
| 76 | Acetic acid, methoxy-, anhydride | 19500-95-9 | Esters | - | ND | 1.52±0.46 | 0.71±0.14 | ND |
| 77 | cis-3-Hexenyl iso-butyrate | 41519-23-7 | Esters | Fruity | ND | 2.04±0.58 | ND | ND |
| 78 | [(*E*)-3-hexen-1-yl butyrate](https://www.chemsrc.com/en/cas/53398-84-8_1628534.html) | 53398-84-8 | Esters | - | ND | ND | 3.35±0.54 | ND |
| 79 | cis-3-Hexenyl-α-methylbutyrate | 53398-85-9 | Esters | Fresh green apple, sweet fruity aroma | 1.1±0.06 | 3.08±0.79 | 3.4±0.79 | ND |
| 80 | 2-Propenoic acid, octyl ester | 2499-59-4 | Esters | - | ND | 3.25±1.05 | ND | ND |
| 81 | *n*-Valeric acid cis-3-hexenyl ester | 35852-46-1 | Esters | Tropical fruity，green | ND | 9.38±1.92 | ND | ND |
| 82 | Hexanoic acid, hexyl ester | 6378-65-0 | Esters | Sweet, fruity, green | ND | 0.74±0.04 | 0.54±0.07 | ND |
| 83 | 4,25-Secoobscurinervan-4-one, O-acetyl-22-ethyl-15,16-dimethoxy-, (22*α*)- | 54658-08-1 | Esters | - | ND | ND | ND | 0.54±0.05 |
| 84 | (*Z*)-3-Hexenyl hexanoate | 31501-11-8 | Esters | Fruity, waxy, green, fatty, winey | 2.61±0.34 | ND | 8.69±2.04 | ND |
| 85 | (*E*)-2-hexenyl hexanoate | 53398-86-0 | Esters | Waxy，green | ND | ND | 1.33±0.23 | ND |
| 86 | (*E*)-Geranic acid methyl ester | 1189-09-9 | Esters | Fruity，waxy | ND | 1.55±0.06 | ND | ND |
| 87 | Methyl 2-methylvalerate | 2177-77-7 | Esters | Tropical fruity | 10.32±1.01 | ND | ND | ND |
| 88 | Benzyl acetate | 140-11-4 | Esters | Fruity, sweet, floral, jasmine | ND | 0.52±0.21 | ND | 1.71±0.26 |
| 89 | [Methyl 2-phenylacetate](https://www.chemsrc.com/en/cas/101-41-7_330257.html) | 101-41-7 | Esters | Spice, waxy, floral, sweet, honey, almond, jasmine odor | ND | ND | ND | 4.17±0.48 |
| 90 | Methyl salicylate | 119-36-8 | Esters | Wintergreen, peppermint | 27.91±1.69 | 114.06±5.22 | 70.64±7.28 | 6.97±1.21 |
| 91 | phenethyl formate | 104-62-1 | Esters | Rose and green hyacinth scent，floral | ND | ND | 0.65±0.19 | ND |
| 92 | 3-hydroxy-2,2,4-trimethylpentyl isobutyrate | 77-68-9 | Esters | - | ND | 0.76±0.07 | ND | ND |
| 93 | Methyl (1-O-retinyl-2,3,4-triacetyl-*β*-D-glucopyran)uronate | 109054-56-0 | Esters | - | ND | ND | 0.24±0.04 | ND |
| 94 | Dimethyl phthalate | 131-11-3 | Esters | Subtle aromatic odor | ND | 0.93±0.17 | ND | ND |
| 95 | 2-Methyl-Butanal | 96-17-3 | Aldehydes | Pekoe scent, malt, sweet, fruity, cocoa | ND | 32.15±1.89 | ND | ND |
| 96 | Pentanal | 110-62-3 | Aldehydes | Pungent odor | ND | ND | 9.82±0.73 | ND |
| 97 | (*E*)-2-Pentenal | 1576-87-0 | Aldehydes | Spicy，green | ND | ND | 4.21±0.1 | ND |
| 98 | Hexanal | 66-25-1 | Aldehydes | Grassy, green, fresh, fatty | 5.88±0.31 | 8.23±1.38 | 5.94±1.99 | ND |
| 99 | (*E*)-2-Hexenal | 6728-26-3 | Aldehydes | Green, leafy, fruity | ND | 30.41±2.34 | 71.71±8.6 | ND |
| 100 | Nonanal | 124-19-6 | Aldehydes | Fatty, citrus, green | 6.57±0.86 | 25.67±1.86 | 30.21±2.68 | 11.87±2.6 |
| 101 | (*E,E*)-2,4-Hexadienal | 142-83-6 | Aldehydes | Green, sweet, fruity, waxy, fatty | ND | ND | 8.05±1.84 | ND |
| 102 | (*E*)-2-Octenal | 2548-87-0 | Aldehydes | Sweet, green, citrus, fatty, herbal, cucumber- | ND | ND | 4.85±0.82 | 2.25±0.73 |
| 103 | (*E,E*)-2,4-Heptadienal | 4313-03-5 | Aldehydes | Stale, fatty | ND | 4.77±0.86 | 4.97±1.8 | 32.29±12 |
| 104 | Benzaldehyde | 100-52-7 | Aldehydes | Green, oily | 12.69±0.59 | 86.04±6.35 | 67.54±7.02 | ND |
| 105 | (*E,Z*)-2,6-Nonadienal | 557-48-2 | Aldehydes | Cucumber-like, violet-like | ND | ND | ND | 4.31±0.41 |
| 106 | *β*-Cyclocitral | 432-25-7 | Aldehydes | Herbal, mint | 1.14±0.39 | 8.28±1.17 | 3.42±0.28 | 6.51±0.68 |
| 107 | Benzeneacetaldehyde | 122-78-1 | Aldehydes | Floral, rose, cherry-like | ND | 99.1±12.43 | 128.06±18.79 | 3.45±0.89 |
| 108 | Safranal | 116-26-7 | Aldehydes | Woody, spicy, herbal | ND | 2.12±0.53 | ND | 2.25±0.41 |
| 109 | (*E*)-2-Decenal | 3913-81-3 | Aldehydes | Green, fatty | ND | ND | ND | 5.81±1.13 |
| 110 | Neral | 106-26-3 | Aldehydes | Sweet lemon flavo、citrus | ND | 4.18±0.27 | 4.55±1.49 | ND |
| 111 | Heptanal | 111-71-7 | Aldehydes | Fatty, green | 12.84±1.71 | ND | 2.16±0.29 | ND |
| 112 | (*E*)-2-Heptenal | 18829-55-5 | Aldehydes | Green, tallow-like | ND | ND | 2.77±0.39 | 7.68±1.11 |
| 113 | (*E*)-Citral | 141-27-5 | Aldehydes | Lemon-like | ND | ND | 12.71±1.78 | ND |
| 114 | Benzeneacetaldehyde, *α*-ethylidene- | 4411-89-6 | Aldehydes | Green, vegetable-like, floral, cocoa-like | ND | ND | 1.08±0.12 | ND |
| 115 | 1-Penten-3-one | 1629-58-9 | Ketones | Spicy | ND | ND | 1.83±0.08 | ND |
| 116 | 3-Penten-2-one, 4-methyl- | 141-79-7 | Ketones | Honey-like, card board-like, nutty, woody | ND | ND | ND | 12.25±2.87 |
| 117 | 14,15*β*-Epoxy-3*β*,11*α*-dihydroxy-5*β*-bufa-20,22-dienolide | 39005-15-7 | Ketones | - | 0.55±0.08 | ND | ND | 0.83±0.18 |
| 118 | 6-Methyl-5-hepten-2-one | 110-93-0 | Ketones | Nutty, fruity | 3.06±0.21 | 10.67±3.01 | 1.99±0.33 | 19.42±2.81 |
| 119 | 3,3',4,4'-Tetradehydro-1,1',2,2'-tetrahydro-1,1'-dimethoxy-2,2'-dioxo-*ψ*,*ψ*-carotene | 1185-31-5 | Ketones | - | 0.43±0.18 | ND | ND | ND |
| 120 | Tetrahydro-2,2,6-trimethyl-6-vinyl-3-pyranone | 33933-72-1 | Ketones | - | ND | ND | ND | 3.03±0.93 |
| 121 | 1-o-Tolylprop-2-en-1-one | 39627-60-6 | Ketones | - | ND | 3.12±0.48 | ND | ND |
| 122 | 3,5-Octadien-2-one | 38284-27-4 | Ketones | Fruity,fatty | ND | 8.9±0.84 | ND | ND |
| 123 | (*E,E*)-3,5-Octadien-2-one | 30086-02-3 | Ketones | Creamy, fruity | ND | 2.37±0.8 | 2.8±0.33 | ND |
| 124 | Acetophenone | 98-86-2 | Ketones | Sweet, cherry-like, vanilla-like | ND | ND | ND | 13.22±1.23 |
| 125 | 2,6,6-Trimethyl-2-cyclohexene-1,4-dione | 1125-21-9 | Ketones | Musty | ND | ND | ND | 1.39±0.45 |
| 126 | Isophorone | 78-59-1 | Ketones | Cooling, woody,sweet, green, fruity | ND | ND | ND | 1.26±0.34 |
| 127 | 2,4,4-Trimethyl-3-(3-methylbutyl)cyclohex-2-enone | 88725-82-0 | Ketones | Sweet, green, fruity | ND | ND | 1.13±0.07 | 3.57±0.49 |
| 128 | Geranylacetone | 3796-70-1 | Ketones | Fresh, rose-like, floral, green, fruity | ND | 2.25±0.2 | ND | ND |
| 129 | (*E*)-*β*-Ionone | 79-77-6 | Ketones | Violet-like, woody | 0.76±0.19 | 10.56±1.06 | 5.43±0.42 | 2.52±0.11 |
| 130 | Jasmone | 488-10-8 | Ketones | Floral, woody, jasmine-like | 1.8±0.51 | 4.85±0.94 | 6.81±0.95 | 0.95±0.21 |
| 131 | [β-Ionone epoxide](https://www.chemsrc.com/en/cas/23267-57-4_263464.html) | 23267-57-4 | Ketones | Fruity,sweet berry aroma，woody | ND | 1.19±0.09 | 0.45±0.11 | 1.52±0.22 |
| 132 | 1,8(2H,5H)-Naphthalenedione, hexahydro-8a-methyl-, cis- | 83406-41-1 | Ketones | - | ND | 7.19±1.53 | ND | ND |
| 133 | *α*-Phellandrene | 99-83-2 | Alkenes | Citrus,terpenic | ND | 2.3±0.61 | ND | ND |
| 134 | 1,3-Cyclohexadiene, 1-methyl-4-(1-methylethyl)- | 99-86-5 | Alkenes | Citrus, woody, spicy, lemon-like | ND | 6.07±0.98 | 1.52±0.45 | ND |
| 135 | D-Limonene | 5989-27-5 | Alkenes | Fruity, lemon-like | 18.31±0.7 | 56.59±10.53 | ND | ND |
| 136 | *γ*-Terpinene | 99-85-4 | Alkenes | Citrus,lemon-like,woody,spicy, | ND | 3.87±0.9 | 1.01±0.02 | ND |
| 137 | Deltacyclene | 7785-10-6 | Alkenes | - | ND | 4.26±1.22 | 2.03±0.23 | ND |
| 138 | Terpinolene | 586-62-9 | Alkenes | Fresh, woody, sweet, piney, citrus | ND | 17.35±2.15 | ND | ND |
| 139 | [Isoterpinolene](https://www.chemsrc.com/en/cas/586-63-0_297673.html) | 586-63-0 | Alkenes | - | ND | ND | ND | 1.34±0.32 |
| 140 | [Bicyclo[3.1.1]hept-2-ene,2-ethenyl-6,6- dimethyl-](https://www.chemsrc.com/en/cas/473-00-7_675370.html) | 473-00-7 | Alkenes | - | ND | 6.59±0.5 | ND | ND |
| 141 | Bicyclo[2.2.1]hept-2-ene, 1,7,7-trimethyl- | 464-17-5 | Alkenes | - | ND | ND | ND | 7.94±1.2 |
| 142 | *β*-Caryophyllene | 87-44-5 | Alkenes | Sweet woody spice， cloves scent，spicy | 3.3±0.5 | ND | ND | ND |
| 143 | *δ*-Cadinene | 483-76-1 | Alkenes | Herbal, woody | ND | ND | 1.8±0.4 | ND |
| 144 | Cyclohexene, 1-methyl-4-(1-methylethenyl)-, (S)- | 5989-54-8 | Alkenes | Terpenic | ND | 34.29±3.73 | ND | 9.4±0.51 |
| 145 | 1-Isopropyl-4,7-dimethyl-1,2,3,5,6,8a-hexahydronaphthalene | 16729-01-4 | Alkenes | Herbal, woody | 3.45±1.02 | 1.87±0.61 | ND | ND |
| 146 | (*E*)-5-Undecene | 764-97-6 | Alkenes | - | ND | 11.42±0.62 | ND | ND |
| 147 | (*Z*)-6-Dodecene | 7206-29-3 | Alkenes | - | ND | 13.63±2.95 | ND | ND |
| 148 | 2-Hexene, 3,5,5-trimethyl- | 26456-76-8 | Alkenes | - | 3.75±1.19 | 6.44±1.63 | 0.83±0.06 | 3.52±0.43 |
| 149 | Myrcene | 123-35-3 | Alkenes | Woody, resinous, musty | ND | 95.09±4.81 | 51.17±5.92 | 5.37±0.35 |
| 150 | (*E*)-*β*-Ocimene | 3779-61-1 | Alkenes | Warm, floral, herbal, sweet | ND | 41.06±5.68 | 15.83±2.83 | ND |
| 151 | (*Z*)-*β*-ocimene | 3338-55-4 | Alkenes | Citrus, herbal, spicy, sweet | ND | 88.3±8.95 | 31.32±4.08 | 3.24±0.43 |
| 152 | Cosmene | 460-01-5 | Alkenes | - | ND | 3.78±0.35 | 3.04±0.32 | ND |
| 153 | 1,4-Hexadiene, 3-ethyl- | 2080-89-9 | Alkenes | - | ND | ND | ND | 2.9±0.52 |
| 154 | 2,4-Octadiene | 13643-08-8 | Alkenes | - | ND | ND | 4.47±0.44 | ND |
| 155 | (*E*)-13-Docosenoic acid | 506-33-2 | Acids | - | ND | ND | ND | 0.89±0.17 |
| 156 | Butanoic acid, 2-methyl- | 116-53-0 | Acids | Pungent，acidic | ND | 8.59±1.12 | 0.87±0.33 | ND |
| 157 | Folic Acid | 59-30-3 | Acids | - | ND | ND | 0.49±0.2 | ND |
| 158 | Pentanoic acid | 109-52-4 | Acids | Fruity | ND | 0.94±0.34 | ND | ND |
| 159 | Hexanoic acid | 142-62-1 | Acids | Fatty, sour, sweat, cheese | ND | 38.84±5.58 | 2.02±0.62 | 2.39±0.49 |
| 160 | (*E*)-3-Hexenoic acid | 1577-18-0 | Acids | Fruity,cheesy | ND | 6.86±0.75 | ND | ND |
| 161 | (*E*)-2-Hexenoic acid | 13419-69-7 | Acids | Fruity | ND | 19.05±3.69 | ND | ND |
| 162 | Nonanoic acid | 112-05-0 | Acids | Green, spicy | ND | 1.16±0.1 | ND | ND |
| 163 | Propanoic acid, 2-methyl- | 79-31-2 | Acids | Yogurt cheese，acidic | ND | ND | ND | 3.2±1.18 |
| 164 | Undecane | 1120-21-4 | Alkanes | - | ND | 34.53±2.69 | ND | ND |
| 165 | Dodecane | 112-40-3 | Alkanes | Alkane | ND | 32.53±3.2 | ND | ND |
| 166 | Octadecane, 3-ethyl-5-(2-ethylbutyl)- | 55282-12-7 | Alkanes | - | 6.25±0.65 | 2.81±0.67 | 0.9±0.19 | ND |
| 167 | Naphthalene, decahydro-1-pentadecyl- | 66359-82-8 | Alkanes | - | ND | ND | 1.58±0.16 | 1.89±0.54 |
| 168 | Phenol | 108-95-2 | Phenols | - | 0.91±0.04 | 1.75±0.31 | 0.65±0.13 | 1.67±0.29 |
| 169 | *p*-Cresol | 106-44-5 | Phenols | Stale, phenolic | ND | ND | ND | 0.6±0.23 |
| 170 | 2,4-Di-tert-butylphenol | 96-76-4 | Phenols | - | 0.77±0.09 | 1.53±0.21 | 0.79±0.22 | 1.11±0.02 |
| 171 | 2(5H)-Furanone, 3-methyl- | 22122-36-7 | Heterocyclics | - | ND | ND | ND | 3.06±0.31 |
| 172 | 2(5H)-Furanone | 497-23-4 | Heterocyclics | Buttery | ND | ND | ND | 2.87±0.55 |
| 173 | [1-(Methylamino)-4-p-toluidinoanthraquinone](https://www.chemsrc.com/en/cas/128-85-8_361816.html) | 128-85-8 | Heterocyclics | - | 0.26±0.05 | ND | ND | 0.26±0.04 |
| 174 | Benzyl nitrile | 140-29-4 | Heterocyclics | - | 1.11±0.18 | 6.88±1.73 | 7.74±0.93 | 1.75±0.06 |
| 175 | Indole | 120-72-9 | Heterocyclics | Floral, animal-like | 0.6±0.03 | ND | 0.28±0.06 | ND |
| 176 | 9-Octadecene, 1-[2-(octadecyloxy)ethoxy]- | 56599-41-8 | Heterocyclics | - | ND | ND | 0.72±0.18 | 0.94±0.08 |
| 177 | Estragole | 140-67-0 | Heterocyclics | Anise | ND | 1.96±0.41 | ND | ND |
| 178 | Anethole | 104-46-1 | Heterocyclics | Sweet fennel， licorice | ND | 0.38±0.04 | ND | ND |
| 179 | 2-Ethylfuran | 3208-16-0 | Heterocyclics | Caramel-like, burnt | ND | 2.87±0.3 | ND | ND |
| 180 | 2-Pentylfuran | 3777-69-3 | Heterocyclics | Nutty flavor | ND | 13.88±2.77 | 1.38±0.12 | ND |
| 181 | (*E*)-2-(2-Pentenyl)furan | 70424-14-5 | Heterocyclics | - | ND | 11.64±1.31 | ND | ND |
| 182 | Furfural | 98-01-1 | Heterocyclics | Sweet, bready, caramel | ND | ND | 1.29±0.15 | 0.69±0.07 |
| 183 | 2-Furanmethanol | 98-00-0 | Heterocyclics | Slight irritating odor，bready | ND | ND | ND | 8.24±1.34 |
| 184 | 1H-Pyrrole-2-carboxaldehyde, 1-ethyl- | 2167-14-8 | Heterocyclics | Burnt, roasted, smoky | ND | ND | 0.34±0.12 | 4.91±0.68 |
| 185 | 4-Hexanolide | 695-06-7 | Others | Caramel, nutty, roasted, sweet | ND | 3.74±0.2 | 0.68±0.07 | ND |
| 186 | Dihydroactinidiolide | 17092-92-1 | Others | Woody | ND | 1.14±0.16 | ND | ND |
| 187 | 2,5-Pyrrolidinedione, 1-ethyl- | 2314-78-5 | Others | - | ND | 2.03±0.89 | 1.13±0.1 | 0.56±0.13 |

Note: GT, green tea; WT, white tea; BT, black tea; DT, dark tea; 'ND' means “Not found”; Odor type derived from websites (http://www.thegoodscentscompany.com; https://www.femaflavor.org; https://www.flavornet.org; https://leffingwell.com) and previous references (Chen et al, 2024; Liu et al., 2022); "-" means the odor type of these volatiles were not obtained;
